# Characteristics and treatment outcomes of malnutrition among infants aged less than 6 months in North–East Nigeria (2019–2022)

**DOI:** 10.1111/mcn.13676

**Published:** 2024-06-04

**Authors:** Nieves Amat Camacho, Faisal Husain, Dang Bahya‐Batinda, Eithandee Aung, Abdullahi Chara, Musa Tanko, Oluwakemi F. Ogundipe, Mario Barbagallo, Kyi Htet Aung, Johan von Schreeb, Francesco Della Corte, Ourania Kolokotroni, Temmy Sunyoto

**Affiliations:** ^1^ Department of Global Public Health, Global Disaster Medicine—Health Needs and Response Karolinska Institutet Stockholm Sweden; ^2^ Center for Research and Training in Disaster Medicine, Humanitarian Aid, and Global Health Università del Piemonte Orientale Novara Italy; ^3^ Médecins Sans Frontières Operational Center Brussels Abuja Nigeria; ^4^ Médecins Sans Frontières, Operational Center Brussels Luxembourg Operational Unit Luxembourg Luxembourg; ^5^ Médecins Sans Frontières Operational Center Brussels Maiduguri Nigeria; ^6^ Médecins Sans Frontières Operational Center Brussels Brussels Belgium; ^7^ Department of Nursing School of Health Sciences Cyprus University of Technology Limassol Cyprus

**Keywords:** ambulatory care, hospitalization, humanitarian assistance, infant malnutrition, Nigeria, severe acute malnutrition, supplementary feeding

## Abstract

Recommendations for the management of malnutrition among infants aged less than 6 months (<6 m) are based on limited evidence. This study aimed to describe the characteristics, treatment outcomes and outcome‐associated factors among malnourished infants <6 m admitted at Médecins Sans Frontières (MSF) inpatient and ambulatory therapeutic feeding centres (ITFC and ATFC) in North–East Nigeria, 2019–2022. We conducted a descriptive analysis of the cohorts and logistic regression to measure the association between two selected outcomes—inpatient mortality and defaulting from the ambulatory programme—and possible factors associated. In total, 940 infants <6 m were admitted at ITFC. Most of them presented severe acute malnutrition and comorbidities, with diarrhoea being the most frequent. On discharge, 13.3% (*n* = 125) of infants were cured, 72.9% (*n* = 684) stabilized (referred to ATFC), 6.5% (*n* = 61) left against medical advice and 4.2% (*n* = 39) died. The median length of hospital stay was 10 days [IQR 7–14]. A hospital stay shorter than 10 days was significantly associated with inpatient mortality (aOR = 12.51, 95% confidence interval [CI] = 3.72–42.11, *p* ≤ 0.01). Among 561 infants followed up at the ATFC, only 2.8% reported comorbidities. On discharge, 80.9% (*n* = 429) were cured, 16.2% (*n* = 86) defaulted and 1.1% (*n* = 6) died. Male sex (aOR = 1.94, 95% CI = 1.15–3.27, *p* = 0.01), internally displaced status (aOR = 1.70, 95% CI = 1.05–2.79, *p* = 0.03) and <−3 WLZ (aOR = 1.95, 95% CI = 1.05–3.63, *p* = 0.03) were significantly associated with programme defaulting. Stabilization and recovery rates among malnourished infants <6 m in the studied project align with acceptable standards in this humanitarian setting. Notable defaulting rates from outpatient care should be further explored.

## Key messages

- We found acceptable stabilization and recovery rates for malnourished infants aged less than 6 months (<6 m) receiving inpatient and ambulatory care in this humanitarian setting.
- There was a considerable proportion of defaulters from the ambulatory programme. Although some potential factors associated with programme defaulting were identified in this study, barriers affecting adherence to ambulatory care should be better explored in the community.
- The factors influencing treatment outcomes among malnourished infants <6 m should be more carefully explored in prospective studies, including additional variables related to infant and maternal characteristics, feeding practices and treatment components.

## Graphical abstract

Study to examine the characteristics, treatment outcomes and outcome‐associated factors among malnourished infants aged less than 6 months admitted at Médecins Sans Frontières Therapeutic Feeding Centers in North–East Nigeria (2019–2022). Stabilization and recovery rates met acceptable standards. Reasons for defaulting from outpatient care should be further explored.

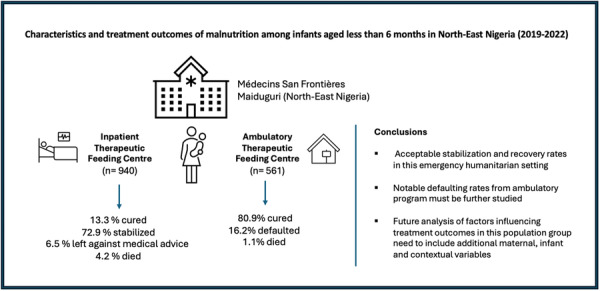

## 1 INTRODUCTION

The burden of malnutrition among infants aged less than 6 months (<6 m) has been increasingly recognized globally. It is estimated that 24.5 million infants <6 m are acutely malnourished (defined as weight‐for‐length [WLZ] <−2 *z*‐score) in 54 low‐ and middle‐income countries (Kerac et al., 2021). About half of the global burden of stunting in early childhood originates during the 500 days between conception and 6 months of age (UNICEF, 2023). Malnutrition among infants <6 m can be triggered by inadequate breastfeeding, but numerous maternal, infant and household risk factors are associated with this complex and multi‐faceted problem (Kerac et al., 2019, 2021; Munirul Islam et al., 2019).

Infants are particularly at heightened risk of malnutrition in low‐income and humanitarian settings, where conditions negatively affect maternal wellbeing, hinder breastfeeding practice and limit access to food and health care services (Al Gasseer et al., 2004; Dall'Oglio et al., 2020; Hirani et al., 2019). Several studies reporting on nutritional programmes operating in humanitarian settings indicate concerns about a high proportion of malnourished infants <6 m identified and highlight challenges associated with their management.(Dureab et al., 2019; Grijalva‐Eternod et al., 2017; Haidar et al., 2017; Oberlin, 2006). The treatment of infants <6 m can be more complex than that of older children, including difficulties in supporting the re‐establishment of breastfeeding (Haidar et al., 2017; Oberlin, 2006) and lack of safe therapeutic feeding options, such as the ready‐to‐use therapeutic food (RUTF) intended for children older than 6 months (Burrell et al., 2020; Munirul Islam et al., 2019).

In 2013, the WHO guidelines for the management of severe acute malnutrition included, for the first time, specific recommendations for infants <6 m. Those—largely based on scarce and low‐quality evidence—were focused on inpatient management and outpatient care was mentioned as a possibility for uncomplicated cases, but without indications about how to practically apply it (World Health Organization, 2013). The WHO guidelines have been updated in 2023, including a special section on the management of infants <6 m at risk of poor growth and development—which encompasses malnourished infants <6 m. The new guidelines incorporate recommendations and good practice statements for programme admission, referral and exit criteria, management of lactation difficulties, milk supplementation and interventions focusing on mothers/caregivers. Yet, the scientific evidence focusing on infants <6 m at risk of poor growth and development remains scarce and of limited quality for most areas, while this paucity of data is especially critical for informing outpatient and community‐based management (World Health Organization, 2023). Exploring approaches to treatment implementation and outcomes, and identifying outcome‐associated factors among infants <6 m and their caregivers is key to closing the evidence gaps and helping with shaping future recommendations.

The North–East region of Nigeria is considered a protracted humanitarian setting, troubled by a long‐standing conflict, internal displacement, disruption of livelihoods and food insecurity, while exposed to drought, flooding and disease outbreaks. Over 1.3 million children under 5 and 152,000 pregnant and lactating women were estimated to be acutely malnourished between January and December 2022 in this region (IPC, 2022). Displacement in this area has been linked to a 57% increase in the likelihood of suffering from acute malnutrition, with notable effects among infants (Iacoella & Tirivayi, 2020). Since 2018, Médecins Sans Frontières (MSF) has run a project in Maiduguri, the capital of Borno State in North–East Nigeria, which focuses on nutrition and also provides outreach health support for internally displaced populations (IDPs). The project provides inpatient and ambulatory care for malnourished children aged 1 month to 10 years, including specific therapeutic management for infants aged <6 m (i.e., 1–5 months).

This study aims to describe the characteristics (demographic, anthropometry, comorbidities) and programmatic outcomes of infants aged 1–5 months admitted at MSF inpatient and ambulatory nutritional programmes in Maiduguri, from 2019 to 2022. It also aims to identify factors associated with selected outcomes (i.e., inpatient mortality and defaulting from the ambulatory programme).

## 2 METHODS

### 2.1 Study design

This is a hospital‐based observational cohort study using previously collected data from an MSF nutrition project in Maiduguri, North–East Nigeria. The study follows the STROBE guidelines for reporting observational studies (STROBE, nd).

### 2.2 Study setting and management of malnourished infants <6 m

Nutritional services for children aged 1 month to 10 years are offered at MSF Nilefa Keji Hospital in Maiduguri, which comprise:

1. Ambulatory Therapeutic Feeding Center (ATFC) managing severely malnourished children without medical complications and following up on malnourished infants <6 m after discharge.
2. Inpatient Therapeutic Feeding Center (ITFC) with 120 bed capacity, including dedicated wards for infants <6 m (around 20 beds).

The criteria for admission and discharge of infants <6 m at ATFC and ITFC in the project, as well as the possible outcomes at discharge from the nutritional programmes, are listed in Table 1. At this facility, all identified malnourished infants <6 m receive inpatient care first; outpatient care is only offered as a follow‐up after hospital admission and discharge.

**Table 1:** ITFC and ATFC admission and discharge criteria for infants <6 m at the MSF facility in Maiduguri (based on MSF protocol and adapted to the project context).

ITFC	ATFC
*Criteria for admission*	
Anthropometrics: Oedema and/or WAZ or WLZ <−2 and/or MUAC <110 mm (≤6 weeks) <115 mm (7 weeks to 5 months) AND/OR either condition affecting feeding or medical complication	Referral for follow‐up after discharge from ITFC
*Criteria for discharge*	
‒ Clinically well ‒ Effective breastfeeding or effective and safe replacement feeding on BMS ‒ Weight gain of approximately 10–20 g/day on exclusive breastfeeding or standard BMS volumes for at least 2–3 days ‒ Absence of oedema ‒ No medical complications or possible to control at home ‒ Caregiver feels prepared for discharge and the family situation is as supportive as possible	‒ WAZ/WLZ ≥−2 on two consecutive visits and MUAC ≥125 mm ‒ Adequate weight gain over at least 2 weeks of approximately 10–20 g/day. ‒ Absence of oedema ‒ Breastfeeding effectively or feeding well with BMS
*Possible outcome*	
Cured: Meets ITFC discharge criteria and achieved WAZ/WLZ ≥−2	Cured: Meet ATFC discharge criteria
Stabilized (ATFC): Meets ITFC discharge criteria but WAZ/WLZ <−2	Disqualified: Wrong assessment of nutritional status, no need for therapeutic treatment
Referral out: Transferred to another facility for advanced medical/surgical treatment	Defaulter: Not attending ATFC for 2 consecutive weeks
Left against medical advice: Leaves the hospital despite medical advice to continue inpatient treatment	Nonrespondent: Exits the programme after 12 weeks of treatment, with stagnant weight, dietary treatment well observed, WLZ >−3 and absence of medical comorbidity
Death: Dies while admitted	Death: Dies within 2 weeks from the last ATFC visit

Abbreviations: ATFC, Outpatient Therapeutic Feeding Centre; BMS, breast milk substitute; ITFC, Inpatient Therapeutic Feeding Centre; MUAC, middle‐upper arm circumference; WAZ, weight‐for‐age *z*‐score; WLZ, weight‐for‐length *z*‐score.

The management of malnourished infants <6 m is essentially focused on enabling and supporting mothers or wet nurses for the establishment of exclusive breastfeeding, which will boost appropriate growth and development. If breastfeeding is not possible, then the infant receives a safe breast milk substitute (BMS). Inpatient nutritional care considers the infant and the mother as one unit, as part of a holistic approach to nutritional care.

The management protocol includes guidance for the stabilization and medical treatment of comorbidities, as well as a nutritional treatment plan. A course of oral antibiotics (i.e., amoxicillin) is routinely administered to all admitted infants. The nutritional treatment comprises three stages: (1) Feeding with diluted F‐100 (Nutriset®) therapeutic milk (or F‐75 if the infant has oedema) providing 100 kcal/kg/day in eight feeds (every 3 h) using a supplementary suckling technique, if possible, and encouraging breastfeeding on demand; (2) If the infant's weight gain is at least 10 g/day, gradual decrease of F‐100 diluted milk quantity to 75%, 50%, 25%; (3) If the infant is gaining 20 g/day on 25% F‐100 diluted milk, change to exclusive breastfeeding on demand and observe if weight gain is maintained. When there is no realistic option of breastfeeding by the mother or wet nurse, infants will start nutritional treatment as in stage 1 and gradually increase supplementation up to 200 kcal/kg/day. Before discharge, the caregiver receives practical health education on how to prepare and give BMS to the infant once at home, safely and appropriately.

In parallel to infant treatment, mothers and wet nurses receive adequate nutrition—a balanced diet and two sachets of RUTF daily—and mental health counselling. Intensive skilled breastfeeding support is provided by midwives and nurses, who are trained following the guidance in the MSF Breastfeeding Booklet. The LATCH tool is used for breastfeeding assessment and the staff provides support for correct positioning and latching, supplementary suckling techniques and strategies to help BF difficulties. In addition to that, nutritional assistants, health promotors and mental health counsellors encourage and provide emotional support to mothers, promoting close skin‐to‐skin contact, breastfeeding on demand and the uptake of recommendations.

During outpatient consultations, infants' weight and length are routinely measured, while occasionally middle‐upper arm circumference (MUAC) is also assessed. Caregivers can report infants' comorbidities or feeding difficulties, and they are referred to health promotors or clinical staff for breastfeeding support or inpatient hospitalization if needed. If the infant is not breastfed, caregivers receive enough quantity of infant formula (Nutribio®) until the next weekly consultation, according to the infant's weight. All lactating mothers or wet nurses receive two sachets per day of RUTF as nutritional supplements during the follow‐up period.

### 2.3 Study participants

The eligible participants for this study were children aged 1–5 months admitted to MSF ITFC and ATFC in Maiduguri from 1 July 2019 until 30 June 2022. There were no exclusion criteria.

### 2.4 Data sources and management

Programmatic data are collected routinely and used to monitor the activities and the overall quality of the services offered. The data were extracted from the existing databases at the MSF project. ITFC data were obtained from a designated Excel database, routinely filled in by data encoders at the MSF facility using the information recorded on each patient file. Patient records at ATFC are directly entered into an electronic application (EasyNut) which can later be transferred to an Excel file. Data management in the project is supervised regularly by the Data Manager who is supported by an epidemiologist covering projects in the country. The ITFC and ATFC databases are not linked (i.e., different patient codes are used) and therefore we present them as two separate cohorts. However, both cohorts contain mostly the same infants since all infants admitted at ATFC were referred for follow‐up after ITFC discharge.

### 2.5 Data analysis

The data were cleaned and analyzed using STATA (version 16; StataCorp LLC). Programme variables considered for analysis included: age (months), sex, residential status, admission date, seasonality (lean [May to August] vs. harvest season [September to April]), anthropometric values on admission (weight, length and MUAC), comorbidities, discharge outcomes and length of stay (days). In the original data set, comorbidities were reported using different terms and including symptoms and signs. For the analysis of the ITFC cohort, we grouped them in categories as follows: diarrhoea/gastroenteritis, respiratory infection, inadequate intake and others specified (which included less frequent conditions reported, such as cardiac disease, anaemia, urinary tract infection, dehydration, hypoglycemia or sepsis) and other nonspecified (i.e., reported as ‘other’ in the original data set). For the ATFC cohort, we left the categorization of comorbidities as they were reported. Weight‐for‐age *z*‐score (WAZ) and WLZ were automatically calculated using reported weight, height, sex and age, based on the 2006 WHO Child Growth Standards.(World Health Organization, 2006) Severe outliers of anthropometric values were excluded following recommendations for anthropometric data cleaning (WAZ <−6 or >5 *z*‐scores and WLZ <−5 or >5 *z*‐scores) (Crowe et al., 2014).

Categorical variables were summarized as counts and percentages, and mean, median and interquartile range (IQR) were calculated for numerical variables. Logistic regression was employed to measure the association between relevant outcomes (dependent variable) and possible explanatory variables (independent). The selected outcomes, considered as dichotomous variables, were mortality at ITFC (death vs. alive at discharge) and defaulting from the ATFC programme (defaulting vs. nondefaulting). The independent variables were infant age, sex, IDP status, WAZ, WLZ, MUAC, comorbidities, seasonality of admission and length of stay. Univariate analysis was undertaken, obtaining crude odds ratio (OR) with 95% confidence intervals (CI) for each variable. A *p* < 0.05 was considered statistically significant. For bivariate analyses, variables exhibiting significant associations were entered into logistic regression models. Adjustment was made for potential confounders, including infant age, sex, IDP status, WAZ, WLZ, MUAC, comorbidities, seasonality of admission and length of stay. Nonsignificant variables were then removed using backwards stepwise elimination, with the least significant variable (*p* > 0.05) removed at each step. This approach allowed us to assess the adjusted associations between the remaining variables and the likelihood of death or defaulting from the ATFC programme, while controlling for potential confounding factors.

### 2.6 Ethical considerations

This study went through an ethical review and was granted an exemption from the Health Research Ethical Committee in Borno State, Nigeria, and the MSF Ethics Review Board for a‐posteriori analyses of routinely collected data. It was conducted with permission from the Medical Director, Operational Center Brussels, Médecins Sans Frontières. The data set was compiled from routine clinical databases and does not include any patient identifying information, to preserve patient confidentiality. A Data Sharing Agreement was signed between MSF and the University of Piemonte Orientale for the purpose of this research.

## 3 RESULTS

### 3.1 ITFC

Infants <6 m accounted for 9.5% (*n* = 940) of children aged 1–59 months admitted to the ITFC during the study period. A description of the cohort is presented in Table 2. The number of admissions increased over time, especially from July 2021. Among admitted infants, 55.8% (*n* = 525) were male and 44.2% (*n* = 415) were female. Median age was 3 months (IQR 2–4). IDPs accounted for 37.5% of admissions.

**Table 2:** Characteristics of infants aged 1–5 months admitted at ITFC and factors associated with inpatient mortality (*n* = 940).

Variables	*N* (%)	OR (95% CI)	*p* Value	aOR (95% CI)	*p* Value
Age (month)					
1	88 (9.3)	1			
2	206 (21.9)	1.0 [0.25–3.96]	0.99		
3	238 (25.3)	1.37 [0.37–5.04]	0.63		
4	182 (19.3)	1.13 [0.28–4.49]	0.85		
5	226 (24.0)	1.45 [0.39–5.34]	0.57		
Sex					
Female	415 (44.2)	1			
Male	525 (55.8)	1.27 [0.66–2.46]	0.46		
Residential status					
Host population	587 (62.4)	1			
Internally displaced	353 (37.5)	1.17 [0.61–2.24]	0.63		
Weight‐for‐age *z*‐score	*n* = *736*				
<−3 *z*‐scores	613 (83.2)	1			
−3 to <−2 *z*‐scores	73 (9.9)	0.75 [0.17–3.27]	0.70		
≥−2 *z*‐scores	50 (6.7)	0.54 [0.07–4.13]	0.55		
Weight‐for‐length *z*‐score	*n* = *690*				
<−3 *z*‐scores	519 (75.2)	1			
−3 to <−2 *z*‐scores	128 (18.5)	1.78 [0.67–4.74]	0.24		
≥−2 *z*‐scores	43 (6.2)	‐	‐		
MUAC	*n* = *387*				
<110	301 (77.7)	1			
110–114	37 (9.5)	1.85 [0.38–8.92]	0.44		
115–125	40 (10.3)	1.70 [0.35–8.19]	0.50		
>125	9 (2.3)	‐	‐		
HIV status					
Positive	12 (1.2)	‐			
Negative	903 (97.1)				
Unknown/not reported	25 (2.6)				
Tuberculosis status					
Negative	789 (87.6)	1		1	
Confirmed or suspected	15 (1.6)	4.50 [0.96–21.01]	0.05	4.19 [0.78–22.60]	0.09
Unknown/not reported	136 (14.4)				
Comorbidities					
Gastroenteritis/diarrhoea	316 (33.6)	1		1	
Respiratory infection	202 (21.4)	1.59 [0.58–4.32]	0.35	1.676 [0.48–5.78]	0.41
Inadequate intake	109 (11.6)	0.35 [0.04–2.88]	0.33	0.737 [0.08–6.51]	0.78
Others specified	92 (9.7)	4.69 [1.79–12.27]	0.00	4.17 [1.13–15.40]	**0.03**
‘Other’ not specified	221 (23.5)	2.22 [0.89–5.52]	0.08	3.15 [1.06–9.38]	**0.04**
Seasonality					
May to August (‘hunger gap’)	422 (44.8)	1			
September to April	518 (55.1)	1.16 [0.61–2.22]	0.63		
Length of stay					
>10 days	517 (55.0)	1		1	
<10 days	423 (45.0)	7.21 [2.9–17.3]	0.001	12.51 [3.72–42.11]	<0.01

Abbreviation: MUAC, middle‐upper‐arm circumference.

Almost all infants (98.5%) were recorded as presenting with a marasmic form of malnutrition. Regarding anthropometry, 204 (21.7%) and 250 (26.5%) cases were excluded when applying data cleaning criteria for WAZ and WLZ values, respectively. Excluding those, the median weight was 3.15 kg (IQR 2.5–3.9) and the median length was 55 cm (IQR 51.5–58.5) on admission. The median MUAC score, encoded for 41.1% of infants, was 100 cm (IQR 88–108). Based on WLZ criteria, 75.2% of infants presented with severe acute malnutrition (<−3 *z*‐score) and 18.5% had moderate acute malnutrition (−3 to <−2 *z*‐score).

Most infants were diagnosed with comorbidities, with diarrhoea/gastroenteritis as the most frequent (33.6%), followed by respiratory infections (21.4%). In 23.5% (*n* = 221) of cases, comorbidities were present but not specified. Through routine screening on admission, 2.8% of infants were diagnosed with malaria, and less than 2% of infants had HIV and tuberculosis.

At discharge, 13.3% (*n* = 125) of infants were recorded as cured, 72.9% (*n* = 684) stabilized (with referral to ATFC), 6.5% (*n* = 61) left against medical advice, 3.1% (*n* = 29) referred to other facilities and 4.2% (*n* = 39) died. Over 82% of deaths occurred after 48h of admission. The mean length of stay among infants who died was 6.2 (SD 3.1) days, and 11.2 (SD 5.18) days for those who survived at discharge. The median length of stay increased over time (Table 3). Some predictors of in‐hospital mortality were identified through logistic regression analysis (Table 2). A hospital admission shorter than 10 days was significantly associated with an increased risk of dying (aOR = 12.51, 95% CI = 3.72–42.11, *p* ≤ 0.01). Presenting conditions included in ‘other specified comorbidities’ (aOR = 4.17, 95% CI = 1.13–15.40, *p* = 0.03) and other non‐specified comorbidities (aOR = 3.15, 95% CI = 1.06–9.38, *p* = 0.04) was also associated with a higher likelihood of death. No other variables were found to be associated with death outcomes.

**Table 3:** Distribution of ITFC and ATFC admissions overtime, discharge outcomes and length of stay.

	1–5 months		1–5 months
	*N* (%)		*N* (%)
ITFC	*n* = 940	ATFC	*n* = 561
**Admission date**		**Admission date**	
Jul 2019 to Jun 2020	242 (25.4)	Jul 2019 to Jun 2020	117 (20.8)
Jul 2020 to Jun 2021	279 (29.6)	Jul 2020 to Jun 2021	140 (24.9)
Jul 2021 to Jun 2022	419 (44.5)	Jul 2021 to Jun 2022	304 (54.1)
**Discharge outcome**		**Discharge outcome**	
Stabilized	684 (72.9)	Cured	429 (80.9)
Referred to other facility	29 (3.0)	Default	86 (16.2)
Cured	125 (13.3)	Disqualified	4 (0.7)
Left against medical advice	61 (6.5)	Transferred	3 (0.5)
Death	39 (4.1)	Nonrespondent	2 (0.3)
Unknown/not reported	2 (0.2)	Death	6 (1.1)
**Length of stay (days) over time**		**Duration of follow‐up (days)**	
*Median (IQR)*	*10 (7–14)*	*Median (IQR)*	*49 (35–69)*
Jul 2019 to Jun 2020	8 (6–11)		
Jul 2020 to Jun 2021	10 (7–14)		
Jul 2021 to Jun 2022	11 (8–15)		

Abbreviations: ATFC, Ambulatory Therapeutic Feeding Center; ITFC, Inpatient Therapeutic Feeding Center.

### 3.2 ATFC

Over the study period, 561 ATFC admissions of infants aged 1–5 months were registered, accounting for 3% of total ATFC admissions from 1 to 59 months at the MSF facility. Infants' characteristics are presented in Table 4. Comorbidities were only reported in 2.8% of cases. In total, 39 (6.9%) and 29 (5.1%) cases were excluded after the cleaning of WAZ and WLZ variables, respectively. On admission, infants had a median weight of 3.5 kg (IQR 3–4.3) and a median length of 54.5 cm (IQR 51–58). MUAC was only measured in 14.9% (*n* = 84) of infants, and the median MUAC score was 108 cm (IQR 95.5–116). A total of 35.9% (*n* = 191) and 33.2% (*n* = 177) of infants presented with severe and moderate acute malnutrition, respectively. WAZ was <−3 *z*‐score in 78.9% (*n* = 412) of infants and the median weight on exit was 4.65 kg (IQR 4–5.3).

**Table 4:** Characteristics of infants aged 1–5 months admitted at ATFC and factors associated with ATFC programme defaulting (*n* = 561).

	*N* (%)	OR (95% CI)	*p* Value	aOR (95% CI)	*p* Value
Age (month)					
1	35 (6.2)				
2	149 (26.5)				
3	153 (27.2)				
4	122 (21.7)				
5	101 (18)				
Unknown/not reported	1 (0.1)				
Sex					
Female	249 (44.3)	1		1	
Male	312 (55.6)	1.49 [1.09–2.92]	0.01	1.94 [1.15–3.27]	0.01
Residential status					
Host	308 (55.0)	1		1	
Internally displaced	252 (45.0)	1.67 [1.05–2.68]	0.03	1.70 [1.05–2.79]	0.03
Unknown/not reported	1 (0.1)				
Weight‐for‐age *z*‐score	*n* = *522*				
≥−2 *z*‐score	46 (8.8)	1			
−3 to <−2 *z*‐score	64 (12.2)	0.61 [0.20–1.85]	0.39		
<−3 *z*‐score	412 (78.9)	0.91 [0.40–2.06]	0.83		
Weight‐for‐length *z*‐score	*n* = *532*				
≥−2 *z*‐score	164 (30.8)	1			
−3 to <−2 *z*‐score	177 (33.2)	1.33 [0.69–2.56]	0.38	1.33 [0.69–2.58]	0.39
<−3 *z*‐score	191 (35.9)	1.93 [1.05–3.57]	0.03	1.95 [1.05–3.63]	0.03
MUAC (mm)	*n* = *84*				
>125	6 (7.3)	1			
115–125	19 (22.6)	1.07 [0.08–12.83]	0.95		
110–114	16 (19.0)	1.15 [0.09–13.87]	0.91		
<110	43 (51.1)	2.60 [0.27–24.94]	0.40		
Comorbidities					
Diarrhoea	4 (0.7)				
Respiratory infection	3 (0.5)				
HIV	4 (0.7)				
Tuberculosis	4 (0.7)				
Malaria	1 (0.1)				

Abbreviation: MUAC, middle‐upper‐arm circumference.

The median duration of follow‐up for infants at the ATFC was 49 days (IQR 35–69). At discharge, 80.9% (*n* = 429) were cured, 16.2% (*n* = 86) defaulted and 1.1% (*n* = 6) died. Logistic regression analysis was done to identify factors associated with defaulting from the ATFC programme. Male sex (aOR = 1.94, 95% CI = 1.15–3.27, *p* = 0.01), IDP status (aOR = 1.70, 95% CI = 1.05–2.79, *p* = 0.03) and <−3 WLZ (aOR = 1.95, 95% CI = 1.05–3.63, *p* = 0.03) were significantly associated with programme defaulting (Table 4).

## 4 DISCUSSION

This study describes the characteristics and treatment outcomes of infants aged 1–5 months admitted at MSF ITFC and ATFC in Maiduguri, North–East Nigeria, from July 2019 to July 2022. It also reports factors associated with two selected programme outcomes: inpatient mortality and defaulting from the ATFC programme.

The results show high stabilization rates among malnourished infants <6 m receiving inpatient care in this protracted emergency setting, and a relatively low mortality (4.1%). A similar in‐hospital mortality was described among malnourished infants <6 m in two previous studies (Grijalva‐Eternod et al., 2017; Munirul Islam et al., 2019), but higher mortality rates were registered in Niger (6%), Yemen (6%) and Kenya (16%).(Baazab et al., 2022; Mwangome et al., 2020; Vygen et al., 2013) ITFC discharge outcomes described in this study were in line with Sphere Standards (Died: <10%; Recovered: >75%; Defaulted: <15%) (Sphere Project, 2018). The use of the supplementary suckling technique for the provision of nutritional treatment has proven to be feasible and effective in this setting. Other studies have also documented positive results with this therapeutic approach (Baazab et al., 2022; Mande et al., 2017; Vygen et al., 2013), despite challenges previously described in the literature (e.g., poor health workers skills, mothers' reluctance) (Lelijveld et al., 2014).

Most infants admitted at ITFC were recorded as having acute malnutrition and comorbidities. Diarrhea was the most common, as in other studies (Baazab et al., 2022; Singh et al., 2014; Vygen et al., 2013). The presence of certain conditions, symptoms and signs, as well as unspecified comorbidities, was identified to be factors associated with death at the MSF ITFC. These results are difficult to interpret with incomplete records of the comorbidities and clinical presentation of infants on admission. Reporting should be improved at the project level to allow the identification of critical clinical risk factors among infants <6 m.

The median ITFC length of stay at this facility (10 days) was longer than the one reported in other studies looking at inpatient care for infants <6 m (Baazab et al., 2022; Mwangome et al., 2020). We observed that ITFC length of stay increased over time in the study setting. This can be explained by progressive efforts to strengthen breastfeeding support in this project, striving for most mothers to achieve exclusive breastfeeding before discharge. The re‐establishment of breastfeeding can be a lengthy process, influenced by the infant's age, previous feeding methods, the mother's motivation and skilled support (Amat Camacho, von Schreeb et al., 2023). Our study showed that the duration of inpatient treatment shorter than 10 days was strongly associated with inpatient mortality, as it was recently reported in a similar study.(Baazab et al., 2022). This finding could be interpreted as a case of reverse causality. Yet, Baazab et al. (2022) suggested that a longer admission time that allows the re‐establishment of effective breastfeeding might contribute to infant survival. In any case, a prolonged length of stay can be inconvenient and have limited acceptance among caregivers, who often leave other children back home while hospitalized. This was portrayed in a qualitative study conducted at this MSF facility, where participants suggested that a long length of stay was problematic and a main reason to default from inpatient care (Amat Camacho, Chara, et al., 2023). The poor uptake of inpatient care was also found to be an important limitation of the treatment effectiveness among infants <6 m with severe malnutrition in Bangladesh (Munirul Islam et al., 2019).

There was a relatively high recovery rate at discharge from the MSF ATFC, where most infants were recorded to be still acutely malnourished on admission. This finding suggests that providing outpatient care for uncomplicated cases—who already established either effective exclusive breastfeeding or artificial feeding—can result in positive outcomes. A study conducted by Mwangome et al. (2020) assessed whether the outcomes achieved through inpatient treatment for malnourished infants <6 m were sustained after discharge—when no outpatient follow‐up was provided. They found that improvements in WLZ and WAZ during hospital admission were generally not maintained beyond 2 weeks post‐discharge, despite 81% of infants leaving the hospital having established exclusive breastfeeding (Mwangome et al., 2020). Outpatient support interventions following inpatient stabilization seem crucial to ensure infant's full recovery after discharge, as recommended in the latest WHO guidelines (World Health Organization, 2023).

In our study setting, all identified malnourished infants <6 m, whether clinically stable or not, were offered inpatient care directly. A few examples of successful community‐based management of mothers with malnourished infants <6 m have been reported from rural Malawi and Senegal (Van Immerzeel et al., 2019; Woeltje et al., 2023). Although this approach might be feasible in some contexts, it seems difficult to be implemented in this study setting, given the instability, displacement and food security deterioration, which already overburden the current nutritional programmes covering this region. Nevertheless, the key features contributing to safe and effective outpatient management of malnourished infants <6 m need to be further investigated in this context.

We identified a considerable proportion of defaulters from ATFC, which was above the acceptable threshold in the Sphere Standards (<15%) (Sphere Project, 2018). Our analysis indicated that infants with worse WLZ anthropometric values were more likely to default, and a similar trend was seen in infants with lower MUAC scores, although the latter was not statistically significant. It could be hypothesized that more severely malnourished infants might have abandoned the programme due to the aggravation of their health status and death in the community. Special attention should also be given to the follow‐up of IDPs since they were also found more likely to drop out of the programme. The reasons behind defaulting among caregivers with infants <6 m should be more closely explored in this context. For instance, looking at the possible logistic difficulties in attending the facility weekly or whether a longer time of admission within the programme due to slower or stagnant weight gain might be contributing factors.

An important proportion of WAZ and WLZ extreme values were flagged during data cleaning, more prominently in the ITFC cohort. The difficulties in obtaining reliable WLZ data in young infants have been previously recognized (Grijalva‐Eternod et al., 2017). Several authors already advocate for considering alternative anthropometry measurements to better identify malnourished infants <6 m more at risk, since MUAC and WAZ appear better associated with mortality than WLZ (Mwangome et al., 2012, 2017). Not all infants admitted at the studied MSF facility had their MUAC measurements recorded because this recommendation was not yet included in guidelines focusing on infants <6 m until its latest 2023 version. Moreover, context‐specific MUAC thresholds have not been determined for this age group (Hoehn et al., 2021). We did not identify any significant association between different WAZ, WLZ or MUAC cut‐offs and mortality with the statistical analysis applied in this cohort. Considering current evidence, and the lack thereof, anthropometric criteria appear to have limited application in this age group. A broader approach to identifying infants <6 m at risk of poor growth and development should be followed, as recommended by the C‐MAMI tool and the latest WHO guidelines, where clinical, feeding and maternal factors should be more critically assessed and considered to guide management actions (ENN et al., 2018; World Health Organization, 2023). Information about these factors could be recorded and encoded in programme databases to allow future comprehensive analyses.

At the study facility, infants <6 m comprised 9.5% of all children aged up to 59 months admitted at the ITFC, and 3% of those followed up at the ATFC. Compared with previous studies, this figure is higher than the proportion of infants <6 m admitted at MSF ITFC found in Niger (3.1%) (Vygen et al., 2013), but lower than the average proportion of young infants reported in a study including different therapeutic feeding programmes in low‐income countries (15.9%) (Grijalva‐Eternod et al., 2017). Our study could not determine whether the burden of malnutrition among infants <6 m reflects the actual prevalence of malnutrition in the MSF project catchment area. However, the latest DHS survey conducted in Nigeria (2018), estimated a 7% prevalence of acute malnutrition (WLZ <−2 *z*‐score) and a 16.8% prevalence of undernutrition (WAZ <−2 *z*‐score) among infants <6 m in the country (ICF, [NPC] Nigeria, 2019). These figures underline the importance of measures to strengthen available support for mothers and infants <6 m at risk of poor growth and development.

Lastly, we observed that the number of overall admissions—including those of children aged more than 6 months—increased overtime, with a substantial rise from July 2021. It should be noted that the location of the project within Maiduguri changed during the summer of 2022, which might be linked to the increase in the number of cases. Nevertheless, our results could also mirror recent reports that highlight the overall deterioration of food security in Nigeria and the consequent increase of children at risk of malnutrition. A United Nations report published in January 2023, estimates that over 25 million people in Nigeria could face food insecurity this year, a 47% increase from the 17 million people who were already at risk of going hungry in the previous year, mainly due to the ongoing insecurity, protracted conflicts and the projected rise in food prices (NFSS, 2022; Save The Children, 2023).

### 4.1 Limitations

This facility‐based study only included malnourished infants <6 m attending the MSF programme, which do not necessarily represent the overall population of malnourished infants in Maiduguri. Moreover, this study used data already collected and encoded in the project databases. This fact hindered the data quality assurance, such as the inaccurate measurement of anthropometric values or lack of systematic reporting of comorbidities and clinical signs. The available encoded data also lacked some relevant variables such as infants' history of prematurity, low birth weight or multiple births, as well as caregivers' characteristics like age, parity, nutritional status or wet nursing. This information is normally registered in patient files but not encoded routinely in the programme databases. Although breastfeeding support is considered a key component in the treatment of malnourished infants <6 m, data on infant feeding practices upon admission and discharge was not recorded in the databases either. Thus, it was not possible to describe breastfeeding difficulties on admission, investigate the change in feeding practices following breastfeeding support or assess the impact of infant feeding on treatment outcomes. Lastly, we could not merge the ITFC and ATFC databases since different patient coding was used in the two databases, although this would have added valuable information about patients' clinical and nutritional progress.

Nevertheless, this study presents results from a relatively large cohort of malnourished infants <6 m receiving inpatient and outpatient care in a protracted emergency setting. The findings are valuable for operational improvement at MSF and contribute to the current scarce knowledge of epidemiology and management of malnutrition among infants <6 m. Future prospective studies should thoroughly investigate infant, maternal and household factors, as these are likely to influence treatment and outcomes of malnutrition among infants <6 m (ENN et al., 2018; Kerac et al., 2021).

## 5 CONCLUSION

The management of malnourished infants <6 m, involving comprehensive inpatient care and outpatient follow‐up for mother–infant dyads, appears to result in stabilization and recovery rates that align with acceptable standards in this humanitarian setting. Particular attention needs to be given to retention in care for outpatient follow‐up. The influence of feeding and maternal and infant characteristics on patient outcomes should be further investigated.

## AUTHOR CONTRIBUTIONS

Nieves Amat Camacho, Temmy Sunyoto and Abdullahi Chara were part of the study design. Musa Tanko and Faisal Husain contributed to data collection and cleaning. Faisal Husain, Dang Bahya‐Batinda, Eithandee Aung, Nieves Amat Camacho and Temmy Sunyoto participated in data analysis. Mario Barbagallo, Kyi Htet Aung, Oluwakemi F. Ogundipe, Johan von Schreeb, Ourania Kolokotroni and Francesco Della Corte revised and contributed to several drafts of the manuscripts. All authors read and approved the final manuscript.

## CONFLICT OF INTEREST STATEMENT

The authors declare no conflict of interest.

## ETHICS STATEMENT

This study went through an ethical review and was granted an exemption from the Health Research Ethical Committee in Borno State, Nigeria (Ref 098/2022) and MSF Ethics Review Board (ref. 2254) for a‐posteriori analyses of routinely collected data.

## Data Availability

Data are available on request in accordance with MSF's data sharing policy. Requests for access to data should be made to data.sharing@msf.org. For more information please see: (1) MSF's Data Sharing Policy: https://www.msf.org/sites/msf.org/files/msf_data_sharing_policycontact_infoannexes_final.pdf (2) MSF's Data Sharing Policy PLOS Medicine article: https://journals.plos.org/plosmedicine/article?id=10.1371/journal.pmed.1001562.
